# Exploring miRNAs’ Based Modeling Approach for Predicting PIRA in Multiple Sclerosis: A Comprehensive Analysis

**DOI:** 10.3390/ijms25126342

**Published:** 2024-06-07

**Authors:** Tommaso Gosetti di Sturmeck, Leonardo Malimpensa, Gina Ferrazzano, Daniele Belvisi, Giorgio Leodori, Flaminia Lembo, Rossella Brandi, Esterina Pascale, Antonino Cattaneo, Marco Salvetti, Antonella Conte, Mara D’Onofrio, Ivan Arisi

**Affiliations:** 1 European Brain Research Institute (EBRI) Rita Levi-Montalcini, 00161 Rome, Italy; t.gosettisturmeck@ebri.it (T.G.d.S.); r.brandi@ebri.it (R.B.); a.cattaneo@ebri.it (A.C.); 2 IRCCS Istituto Neurologico Mediterraneo Neuromed, 86077 Pozzilli, Italy; leonardo.malimpensa@uniroma1.it (L.M.); daniele.belvisi@uniroma1.it (D.B.); giorgio.leodori@uniroma1.it (G.L.); marco.salvetti@uniroma1.it (M.S.); antonella.conte@uniroma1.it (A.C.); 3 Department of Human Neurosciences, Sapienza University of Rome, 00185 Rome, Italy; gina.ferrazzano@uniroma1.it (G.F.); lembo.1844672@studenti.uniroma1.it (F.L.); 4 Department of Medico-Surgical Sciences and Biotechnologies, Sapienza University of Rome, 04100 Latina, Italy; esterina.pascale@uniroma1.it; 5 Bio@SNS Laboratory of Biology, Scuola Normale Superiore (SNS), 56126 Pisa, Italy; 6 Centre for Experimental Neurological Therapies (CENTERS), Department of Neurosciences, Mental Health and Sensory Organs, Sapienza University of Rome, 00189 Rome, Italy; 7 Institute of Translational Pharmacology, National Research Council, 00133 Rome, Italy

**Keywords:** progression independent of relapse activity (PIRA), multiple sclerosis, modeling approach, miRNA

## Abstract

The current hypothesis on the pathophysiology of multiple sclerosis (MS) suggests the involvement of both inflammatory and neurodegenerative mechanisms. Disease Modifying Therapies (DMTs) effectively decrease relapse rates, thus reducing relapse-associated disability in people with MS. In some patients, disability progression, however, is not solely linked to new lesions and clinical relapses but can manifest independently. Progression Independent of Relapse Activity (PIRA) significantly contributes to long-term disability, stressing the urge to unveil biomarkers to forecast disease progression. Twenty-five adult patients with relapsing–remitting multiple sclerosis (RRMS) were enrolled in a cohort study, according to the latest McDonald criteria, and tested before and after high-efficacy Disease Modifying Therapies (DMTs) (6–24 months). Through Agilent microarrays, we analyzed miRNA profiles from peripheral blood mononuclear cells. Multivariate logistic and linear models with interactions were generated. Robustness was assessed by randomization tests in R. A subset of miRNAs, correlated with PIRA, and the Expanded Disability Status Scale (EDSS), was selected. To refine the patient stratification connected to the disease trajectory, we computed a robust logistic classification model derived from baseline miRNA expression to predict PIRA status (AUC = 0.971). We built an optimal multilinear model by selecting four other miRNA predictors to describe EDSS changes compared to baseline. Multivariate modeling offers a promising avenue to uncover potential biomarkers essential for accurate prediction of disability progression in early MS stages. These models can provide valuable insights into developing personalized and effective treatment strategies.

## 1. Introduction

Multiple sclerosis (MS) is a chronic autoimmune disease affecting the central nervous system, triggering a wide range of symptoms resulting in cognitive, motor, sensory, sphincteric, and visual function impairments [1]. MS is the most widespread source of non-traumatic neurological disability in young adults, with an incidence of around 1 per 1000 [2]. Furthermore, MS displays a significant social and economic burden on patients, their families, and society, with substantial costs beyond direct healthcare expenses, including reduced quality of life and the emotional toll on caregivers [3]. MS patients accumulate progressive disability due to chronic inflammation and neurodegeneration. In some patients, neurodegeneration may develop early in the disease process and represents a driver of disease progression [4,5,6]. It is generally felt that the existing classification of MS in different subtypes does not reflect the clinical and biological heterogeneous nature of the disease [4]. Although many DMTs are now available [7,8,9], the choice and sequencing require a personalized approach. Treatment selection is also based on individual demographic and clinical factors such as MS-related prognostic factors, patient comorbidities, risk tolerance, pregnancy planning, and route of administration [8].

Recent evidence has emphasized the effectiveness of high-efficacy-DMTs in reducing relapse rates, inflammation foci, and slowing down the relapse-associated accumulation of disability over time [10]. Current research indicates, however, that disability progression in MS patients is not solely linked to new focal inflammatory demyelinating lesions and clinical relapse [11]. Instead, it is increasingly recognized that progression independent of relapse activity (PIRA) [12,13,14] and the accumulation of disability in the absence of relapse-associated worsening (RAW) [12], as determined by the Expanded Disability Status Scale (EDSS), may occur from the disease onset [10,15]. In the early stages of MS, PIRA is a significant provider of long-term disability, even in the absence of relapses [16,17]. Relapsing multiple sclerosis (MS) appears to be orchestrated by the activation and migration of peripheral immune cells into the central nervous system (CNS), with a compelling focus on the interplay between T and B cells. Non-relapsing progressive MS seems to be related to neurodegeneration phenomenon and/or smoldering CNS inflammation [18,19]. PIRA has been associated with several MRI features, including brain and spinal cord atrophy, as well as an increase in paramagnetic rim lesions [20,21]. The pathophysiological substrate for PIRA is currently not extensively characterized, with a combination of pathological processes probably contributing to degenerative progression in early MS [20,22,23,24]. Moreover, studies have highlighted the challenges of compensatory mechanisms to leptomeningeal inflammation failure and focal spinal cord pathology, which are potentially linked to PIRA [25]. To date, the clinical and neuroimaging predictors of PIRA at disease onset have not yet been outlined, nor is its association with inflammation [16,26]. As a result, PIRA has gained significant attention in both research and clinical settings. Given that patients experiencing PIRA early in the disease course often face a challenging prognosis, there is a pressing need to unveil biomarkers capable of predicting and monitoring the clinical evolution of MS and the response to various DMTs.

Among potential biomarkers, miRNAs have gained significant consideration over time due to their role in gene expression regulation at the post-transcriptional level. Furthermore, miRNAs’ involvement in diverse cellular processes, such as inflammation, neurodegeneration, and remyelination, has been extensively investigated [27,28]. miRNAs are small, non-coding RNA molecules, between 20 and 25 nucleotides, which regulate a multitude of cellular processes. Different signatures of miRNA expression have been identified in MS subjects in the relapse versus remitting phase, able to identify specific drug responses and radiological patterns [29,30,31]. A specific signature of deregulated miRNAs in peripheral blood mononuclear cells (PBMC) has been shown as a biomarker in disease stratification or DMT response [32].

Modeling approaches, such as logistic and multilinear regressions are essential in modern medicine for predicting outcomes and understanding complex relationships between variables. These statistical tools enabled researchers using a backstep multivariate regression analysis to identify miRNAs significantly correlated with EDSS changes over time in MS patients [33,34,35].

As of today, there are limited notions regarding biomarkers-based models as potential predictors of MS disease progression. The study aimed to identify miRNAs associated with MS progression by analyzing their expression levels in relation to PIRA and EDSS scores. MiRNAs correlated with PIRA status in MS patients were highlighted using a predictive probabilistic regression model to accurately stratify future PIRA risk. A multivariate analysis further revealed miRNA predictors associated with changes in EDSS scores, demonstrating the utility of regression in forecasting disability progression.

## 2. Results

### 2.1. Patient Screening and Enrollment

Twenty-five adult patients with relapsing–remitting MS (RRMS) (17 females and 8 males) were enrolled in this study, according to the latest McDonald criteria [9]. Patients underwent clinical assessments before (time T0) and after high efficacy DMTs at different time points (6, 12, 18, 24 months: T1, T2, T3, T4, respectively). Patients’ demographic and main clinical features are reported in Table 1. RAW was defined as a confirmed and sustained disability worsening (CDW) event with an onset within 90 days from the beginning of a relapse. PIRA was defined as a CDW event either without any preceding relapse or with an onset occurring more than 90 days after the beginning of the reported relapse. Peripheral blood was collected at baseline (T0) to measure miRNA expression profiles.

### 2.2. Identifying Candidate miRNAs Linked with Multiple Sclerosis Progression

**Figure 1:**
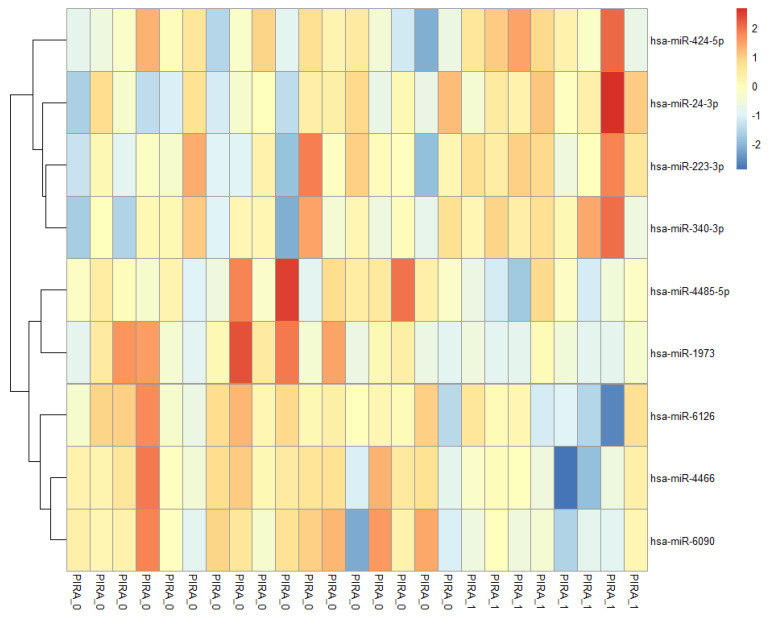
Sample heatmap visualization. The heatmap plot is based on Log2 normalized and standardized expression data (zero-centered, SD = 1.0) of the 9 miRNA genes significantly correlated with PIRA status (0/1). Samples are labeled according to PIRA.

**Figure 2:**
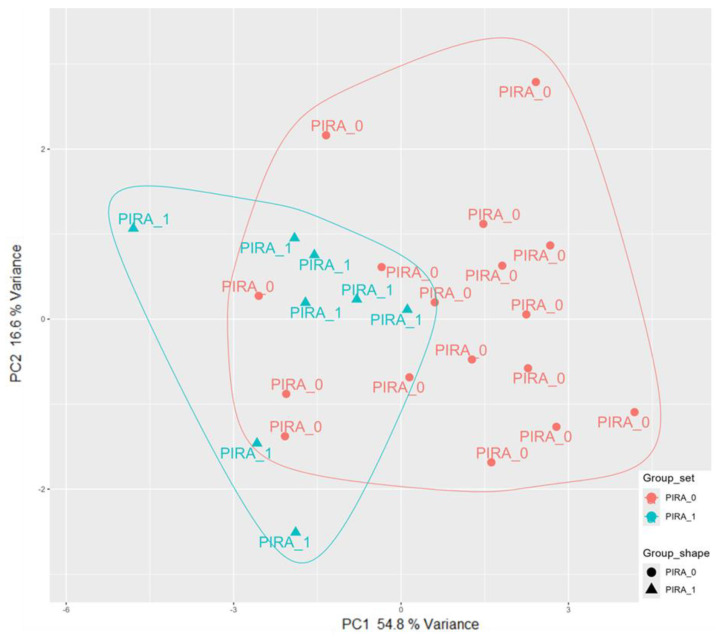
Principal component analysis of samples. The PCA plot is based on Log2 normalized expression data of the 9 miRNA genes significantly correlated with PIRA status and EDSS score change. Samples are labeled according to PIRA.

With the aim of designing an optimal model to associate miRNA expression levels to clinical trajectory as quantified by PIRA and EDSS score, we performed a first filtering step to select miRNAs with significant correlation to PIRA status (0/1). This resulted in a pre-selection of nine miRNA genes (hsa-miR-4485-5p, hsa-miR-1973, hsa-miR-424-5p, hsa-miR-4466, hsa-miR-6126, hsa-miR-223-3p, hsa-miR-24-3p, hsa-miR-340-3p, hsa-miR-6090) (Table 2), shown in a heatmap (Figure 1). The Principal Component Analysis (PCA) highlighted how these genes collectively could partially stratify the subjects based on PIRA (Figure 2).

**Figure 3:**
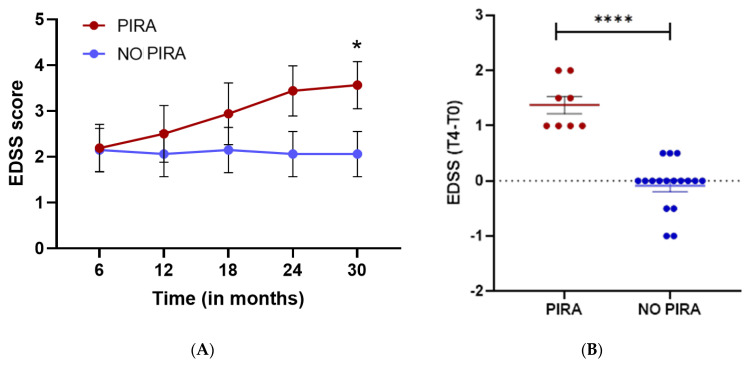
EDSS score variation and trajectory based on PIRA. (**A**) EDSS trajectory based on PIRA status. Subjects are divided according to the PIRA status and compared using the Mann–Whitney two-sided test. (*) *p* < 0.05. (**B**) EDSS score variation between T4 and T0 time points. Subjects are divided according to the PIRA status. The two groups are compared using the Mann–Whitney two-sided test. (****) *p* < 0.0001.

The trajectory of EDSS scores in subjects was influenced by the stratification of PIRA, with this relationship becoming increasingly visible at later disease stages (Figure 3A). Moreover, the change in EDSS score at T4 compared to T0 (EDSS_T4_ − EDSS_T0_) differed according to PIRA status, with a significantly larger deviation from baseline when PIRA was present (Figure 3B).

### 2.3. Developing a Predictive Model for PIRA

In order to refine patients’ stratification, which is tightly connected to the EDSS score trajectory, we looked for a robust logistic classification model constructed on four baseline (T0) miRNA expression levels and their first-order interactions, to predict the PIRA status at follow-up (0 = No PIRA, 1 = PIRA). The selected optimal logistic score-based model allowed both a good prediction for PIRA status, based on score cut-off, and had statistically significant coefficient values (Equation (1)).

(1) $$Logistic\_score=\frac{e^{Z}}{1+e^{Z}}\;PIRA\_status=\left\{\begin{array}{c} 0\to No\;PIRA,\;\;\;if\;Logistic\_score<0.277\\ 1\to PIRA,\;\;\;\;\;\;\;\;\;if\;Logistic\_score\geq 0.277 \end{array}\right.$$

where

*Z* = −425.78 + 43.66*miR-4485-5p + 154.79*miR-340-3p − 20.42*miR-6126*miR-4485-5p + 15.46*miR-223-3p*miR-6126 + 15.59*miR-340-3p*miR-6126 − 9.49*miR-340-3p*miR-223-3p.

**Figure 4:**
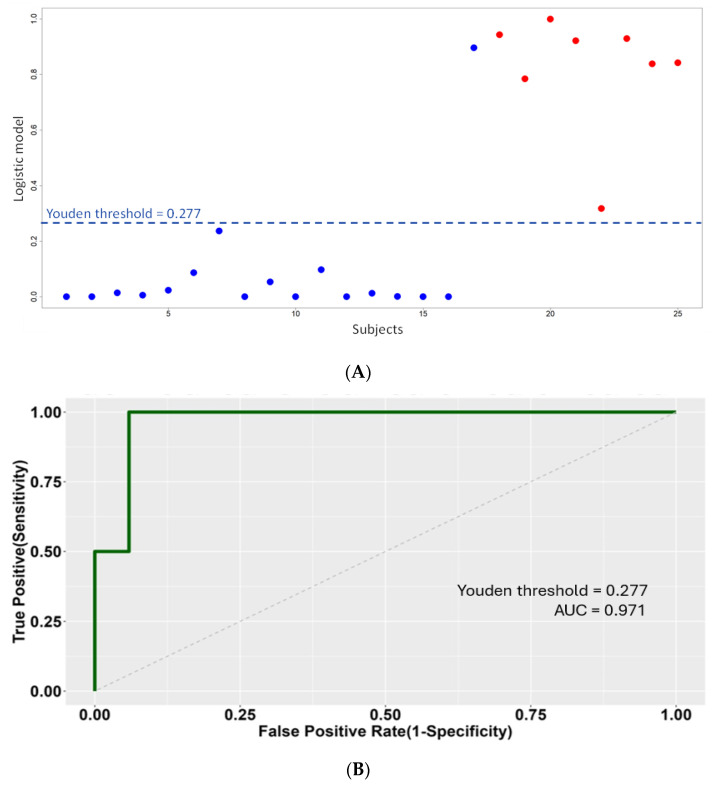
(**A**) Predicted scores obtained by the logistic model. The horizontal dashed line corresponds to the cut-off, estimated as the maximum Youden index in the corresponding ROC curve, between the two levels 0/1 = negative/positive of PIRA status based on actual clinical data. Negative PIRA subjects in clinical data are plotted in blue and similarly positive ones in red. All model coefficients, including intercept, are significant (Wald test, *p* < 0.05); see Equation (1). (**B**) ROC curve of the binary classifier logistic model. The AUC is significantly large, according to reference null distributions obtained by randomizing PIRA status (*p* < 0.01) or miRNA predictors (*p* < 0.05); see Section 4.

A score of 0.277 = optimal threshold of the logistic score by the maximum Youden method in the ROC curve (Figure 4).

All model coefficients, including intercept, were statistically significant (Wald test, *p* < 0.05).

Using this model, logistic scores were computed, together with the corresponding ROC curve. The model is also able to predict the correct PIRA status, with a computed AUC = 0.971 (bootstrapped AUC = 0.990 ± 0.023 with a high degree of similarity). The maximum Youden index criterion allowed to select an optimal score cut-off = 0.277 to discriminate between positive (>cut-off) and negative (<cut-off) PIRA status. The robustness of AUC was assessed by randomization tests on reshuffled PIRA status and predictor identity (Figure 4A,B).

### 2.4. Evaluating EDSS Changes with a Multivariate miRNA Analysis

Using four other miRNA predictors associated with EDSS, it was possible to build an optimal multilinear model, including interactions, to describe the relative EDSS changes after 24 months (*EDSS_T_*_4_ − *EDSS_T_*_0_, Equation (2)).

*EDSS*_*T*4_ − *EDSS*_*T*0_ = 1183.40 + 75.06*miR-4466 − 185.95*miR-24-3p − 159.92*miR-6090 + 11.97*miR-223-3p*miR-4466 − 23.99*miR-24-3p*miR-4466 + 4.76*miR-24-3p*miR-223-3p − 14.80*miR-6090*miR-223-3p + 36.84*miR-6090*miR-24-3p. (2)

All model coefficients, including intercept, were again statistically significant (Wald test, *p* < 0.05).

**Figure 5:**
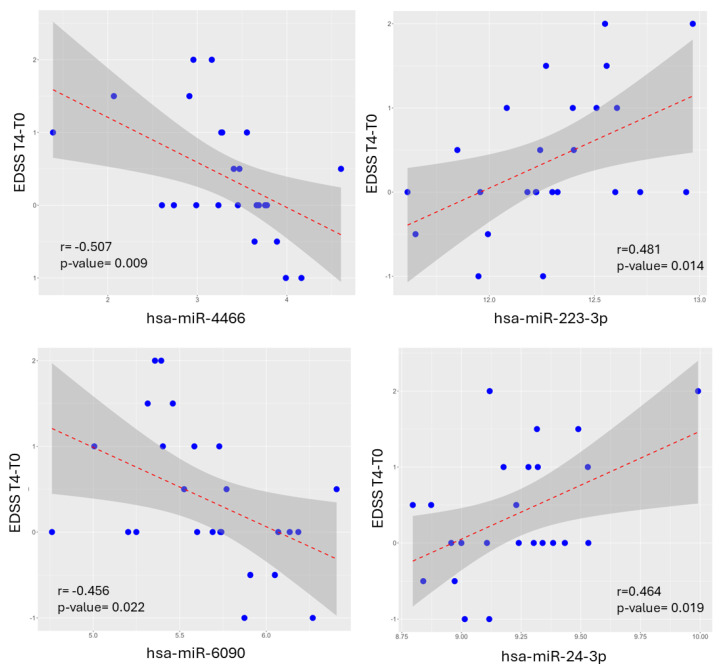
Correlation plots between selected miRNAs and EDSS (T4-T0). The four miRNAs used for the multilinear regression model in Equation (2) are significantly correlated to EDSS change at T4. The linear regression line in red is surrounded by the confidence interval in dark gray. MiRNA and EDSS data are normally distributed (Shapiro–Wilk test and Kolmogorov–Smirnov test, *p* < 0.05).

Though each one of these four miRNA genes were significantly correlated with the EDSS changes at T4 (Figure 5), none singularly could be used for an optimal univariate regression model, since the performance was consistently worse than the full multivariate model in Equation (2) combining both single predictors and their interactions.

**Figure 6:**
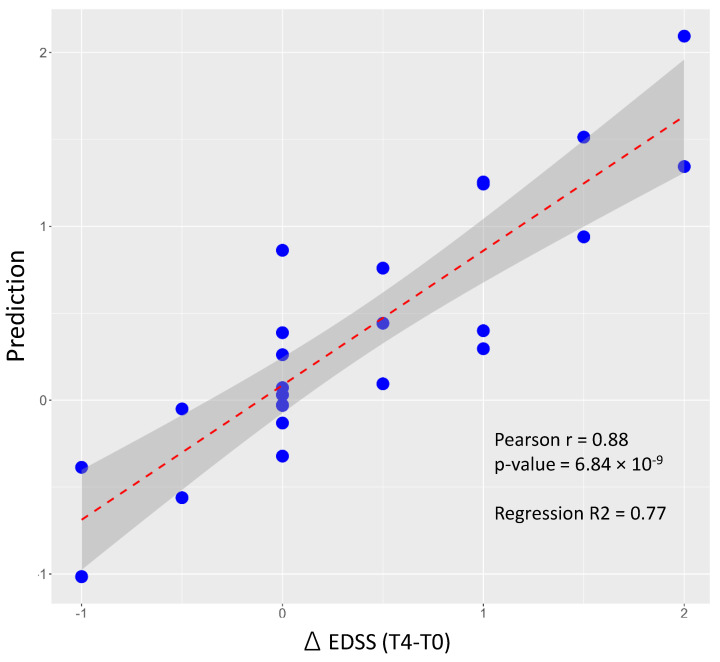
Multilinear model to predict EDSS (T4-T0). The multilinear model is based on the same four miRNA predictors used for the logistic model. The gray band represents the confidence interval around the linear regression line in red. The Pearson correlation between actual data and prediction is significant (*p* < 0.00001); see Equation (2). Prediction values and EDSS data are normally distributed (Shapiro–Wilk test and Kolmogorov–Smirnov test, *p* < 0.05).

The multivariate model performance in predicting changes in EDSS compared to baseline is shown in Figure 6, with a very good correlation level between predicted and real data.

## 3. Discussion

Accurately forecasting the trajectory of multiple sclerosis is a critical medical need, as predicting disease progression can equip clinicians to empower treatment interventions in a timely manner and improve patient outcomes. This study aimed to investigate whether miRNA baseline expression profiles in PBMC predicted disability worsening due to PIRA in RRMS patients. We designed logistic models to associate miRNA expression to clinical trajectory as quantified by PIRA, a binary variable, and EDSS scores. We decided to use multivariate logistic and linear models, including interaction terms, to improve the regression quality. We first pre-selected nine miRNA genes (hsa-miR-4485-5p, hsa-miR-1973, **hsa-miR-424-5p**, hsa-miR-4466, hsa-miR-6126, **hsa-miR-223-3p**, **hsa-miR-24-3p**, **hsa-miR-340-3p**, hsa-miR-6090), significantly correlated to PIRA which were also correlated to EDSS changes (T4-T0) (Table 2). This first step was necessary in order to perform a preliminary feature selection to narrow the set of predictor combinations for model building.

The PCA of samples (Figure 2) obtained using these nine potential predictors highlighted how miRNAs partially discriminated between positive and negative PIRA conditions, but a further optimization step was required. The EDSS trajectories of subjects, as the disease severity increased, strictly depended on the PIRA stratification (Figure 3). No studies are available in the literature that clearly associates miRNA expression levels with PIRA. Hence, we were unable to benchmark our model with other results, nor were we able to validate the model using an independent dataset, as miRNA data collection is not a common practice in everyday clinical activities. We constructed the logistic formula taking care by selecting an optimal model with statistically significant coefficients for reasonable robustness. Additionally, we assessed the AUC significance using two different empirical null distributions, which were obtained by randomizing the PIRA status or by randomly selecting unrelated miRNA predictors.

Different authors investigated the link between miRNA expression and EDSS in serum. Using a backstep multivariate regression analysis, Casanova et al. identified hsa-miR-9-5p as significantly correlated with EDSS change over 24 months, mirroring the timeframe of our research [34]. Conversely, unlike our approach, they did not construct a multivariate model to derive an optimized score associated with EDSS changes over time. Nevertheless, consistent with the analysis approach in the current study, prior research has found that single miRNA predictors were unable to achieve optimal performance in univariate logistic models. In contrast, multivariate logistic models were able to attain better predictive performance [36,37]. However, many of the model coefficients in these multivariate analyses did not reach statistical significance. This highlights the challenge of identifying robust miRNAs as biomarkers for multiple sclerosis disease staging using a univariate approach. The higher number of studies using multivariate models suggests the need to consider the combined effects of multiple miRNAs rather than relying on individual miRNA predictors alone. The lack of statistical significance for some model coefficients underscores the complexity of establishing definitive associations between specific miRNAs and disease staging in multiple sclerosis. Based on our findings, we advance the hypothesis that the nine chosen miRNAs may have potential associations with both PIRA status and EDSS change during 24-month follow-up. Additionally, a stronger and more comprehensive association could potentially be achieved through the implementation of appropriate multivariate logistic modeling for PIRA and linear modeling for EDSS. A more robust understanding of the relationships between miRNA expression, PIRA, and EDSS increase and progression could be achieved by integrating a specific subset of predictors along with their multiplicative interaction terms.

Furthermore, with the logistic model being indicated as a possible marker of progression in this study, some of the microRNAs identified and correlated with PIRA and EDSS appear to have specific associations with the pathophysiology of multiple sclerosis based on the literature. Some miRNAs have been recently proposed as biomarkers for RRMS in serum [34] and extracellular vesicles (EVs) [35,38,39]. In the present study, we showed that miRNA expression levels were associated with PIRA. MiR-223-3p has a pivotal role as an anti-inflammatory in immune cells and serves as a suppressor of NLRP3, a key protein of the inflammasome, recognized as a central component in the development of several inflammatory and autoimmune diseases [40]. NLRP3 inflammasome activity is also critical for the inflammation-based microenvironment following demyelination and is a potential therapeutic target for inflammatory-mediated demyelinating diseases, including MS [41]. The overexpression of miR-24-3p [42] and the downregulation of miR-223-3p [43] have already been shown in RRMS, and the correlation with EDSS has been reported [34,44]. Moreover, Scaroni et al. in 2022 found both miR-223-3p and miR-24-3p overexpressed in serum EVs in CI (cognitively impaired) MS patients when compared to CP (cognitively preserved) MS patients [38]. Vistbakka et al., 2022 described miR223-3p variation in all the subtypes studied across a 4-year follow-up but without a clear correlation with the clinical disability, as measured by EDSS. Over the same follow-up period, the expression of miR-24-3p was stable longitudinally, while miR-223-3p resulted in temporary variation [42]. In agreement with a previous study, we found that miR-24-3p correlated with the disability progression in RRMS. Our findings confirm the temporal correlation of miR-223-3p and miR-24-3p with clinical disability as measured by EDSS in RRMS.

Moreover, some of the miRNA in our model, such as miR-340-3p and miR-424-5p, have a potential role in inflammation and in MS. MiR-340-3p is involved in the inflammatory processes and is reduced in B cells of RRMS patients and able to induce a specific cytokine and chemokine response [45]. MiR-340-3p has been described by Wallach et al. as a novel TLR7/8 activator involved in CNS injury, thereby providing its potential role as a signaling molecule in CNS diseases. **MiR-424-5p** has been identified in the plasma of subjects who remained as Radiologically Isolated Syndrome (RIS) after 5 years of follow-up [46].

We propose a multivariate-based approach model that can explore the association between miRNAs and clinical activity and progression in MS. This model, based on measures of disability, such as the EDSS and PIRA, could uncover potential biological biomarkers essential for accurately predicting disability progression in the early stages of the disease.

While these findings are promising, the lack of an independent validation cohort and the complexity of establishing definitive associations between specific miRNAs and disease staging in MS highlights the need for further research on a larger patient subset. Expanding the analysis by including a broader range of variables may yield additional insights into MS disease progression mechanisms and provide a more robust and reliable disease trajectory modeling. To elucidate and investigate the complex pathophysiology of MS and the specific response to DMTs, a comprehensive approach is further needed. This probably would involve the integration of biomarkers, such as miRNAs, from diverse sources, including PBMC, serum, EVs, and single-cell technologies, through modeling strategies that can capture disease progression course. If these miRNA-based models were validated and optimized in a larger, well-characterized population, they would unlock the potential for targeted pharmacological interventions in preventing disease progression.

## 4. Materials and Methods

### 4.1. Study Design and Participants

Patients with RRMS, according to the latest McDonald revised criteria [9], were enrolled. DMTs have been chosen as indicated [47]. The patients were clinically tested before (time 0 = T0) and after treatment (T1–T4, 6–24 months). To protect sensitive data, anonymous codes were assigned to each participant and preserved for the study duration. All subjects gave written informed consent to participate in the study. The research was conducted following the Helsinki Declaration and approved by the Ethics of Sapienza University-Policlinico Umberto I (Rif. 6361, protocol number 0635/2021). To minimize potential bias factors, all clinical data were gathered in the same clinical center following the same guidelines.

### 4.2. PBMC Sample Collection

The patient’s peripheral blood was drawn via venipuncture at T0 for miRNAs’ profiling and other laboratory tests. The blood samples were collected in Vacutainer tubes containing EDTA. Then, 15 mL of phosphate-buffered saline (PBS; without Ca^2+^, Mg^2+^) was added to 10 mL of each sample’s blood; after mixing, the diluted blood samples were carefully layered onto 7.5 mL of Ficoll for 30 min of centrifugation (18–20 °C) at 1800 rpm. The lymphocytes/monocytes layer was accurately collected in clean tubes; the cells were then pelted (1400 rpm for 10 min at 18–20 °C) and washed with PBS. The dry pellet was finally stored at −80 °C.

### 4.3. RNA Extraction and Quality Checked

RNA extraction and quality control RNA extraction was performed according to the miRNeasy Tissue/Cells Advanced Mini Kit (QIAGEN, Redwood City, CA, USA) instructions; the cells were suspended in 500 mL of RTL buffer + b mercaptoethanol, incubated at 37 °C for 10 min, and homogenized. The samples were passed through two different spin columns: the gDNA Eliminator spin column to remove all DNA and the Rneasy spin column to select RNA molecules. The miRNeasy Tissue/Cells Advanced Kits enabled efficient RNA enrichment down to approximately 18 nucleotides in size. All RNA samples were again stored at −80 °C. RNA purity and concentration quality control included the evaluation of absorbance at 260 nm by NanoDrop ND-1000 (Labtech International, Ringmer, UK). To assess the RNA integrity, samples were tested in the Agilent 2100 Bioanalyzer (Agilent Technologies, Santa Clara, CA, USA) via the Eukaryote Total RNA 6000 Nano kit (Agilent Technologies, Santa Clara, CA, USA) and the Small RNA kit (Agilent Technologies, Santa Clara, CA, USA). The bioanalyzer assessed each sample’s RNA integrity number (RIN). Samples displaying an under-threshold RIN value (<8.0) were excluded from analysis.

### 4.4. Agilent Microarray miRNA Profiles

The miRNA profiles were performed according to the standard Agilent miRNA Microarray protocol (Agilent Technologies, Version 3.1.1, 2015, Santa Clara, CA, USA). After a phosphatase treatment and a denaturation process via DMSO, 100 ng of RNA extracted from each sample was labeled with 3-pCp cyanine. Samples were hybridized to the Agilent Human miRNA Microarrays chip 8 × 60 K (Agilent PN G4870-60530, grid ID = 070156) containing 2549 human miRNAs. The glasses were incubated in the Agilent Hybridization Oven at 55 °C, 10 RPM, for 20 h, washed according to the protocol, and scanned using the Agilent DNA Microarray Scanner (G2539C).

### 4.5. Statistical Analyses

MiRNA expression values are median normalized and Log2 transformed. Only miRNAs with expression values > 0.0 in every sample were included in the analysis. Data analysis was performed using R-Bioconductor [48,49]. Pearson’s correlation coefficient was used to estimate the association between miRNA profiles and clinical measures. The Shapiro–Wilk test was first applied to verify that the sample data followed a normal distribution, with a *p*-value > 0.05 indicating the data was consistent with a normal distribution [50]. Only miRNAs with statistically significant correlation (*p* < 0.05) were selected for further analysis. Multivariate logistic and linear models were generated by the R package glmulti [51], including binary interaction terms. We selected the best model with 4 miRNA predictors out of 9 miRNA genes significantly correlated with PIRA. The statistical significance of coefficients was assessed using the Wald test (null hypothesis coeff = 0). ROC curves were obtained by the R package ROCR [52] and plotted by ggplot2 [53]. Heatmaps were obtained by pheatmap [54]. The robustness of AUC for the optimal logistic model was assessed by 1000 bootstrapped resampling of data and by two different randomization tests: 1000 randomizations of binary logistic response variable (PIRA) and 10,000 randomizations of miRNA predictors by randomly selecting non-optimal or unrelated miRNAs to evaluate the same model, so creating empirical null distributions. Experimental groups were compared using the Mann–Whitney two-sided test.

## Figures and Tables

**Table 1:** Epidemiological and clinical data of the enrolled patients. Values are expressed as the number of subjects or (mean ± SD) otherwise.

Data Entry	Value
Sex (M/F)	M = 8, F = 17
Age	43 ± 8.9
Disease Duration (years)	9.48 ± 8.42
Relapse 1 year before DMT treatment (Yes/No)	Y = 22, N = 3
Relapse post DMT first treatment (Yes/No)	Y = 3, N = 22
RAW (relapse-associated worsening, Yes/No)	Y = 3, N = 22
PIRA (progression independent of relapse activity, 1 = Yes/0 = No)	Y = 8, N = 17
EDSS T0 (baseline)	2.2 ± 1.78
EDSS T4 (24 months)	2.54 ± 1.97

**Table 2:** Correlation values between selected miRNAs, PIRA status, and EDSS (T4-T0) difference. Nine miRNAs were selected based on their significant correlation both with PIRA and EDSS change at 24 months (T4). These miRNAs were then used to build logistic and multilinear models. All correlations are statistically significant (*p* < 0.05).

	PIRA		EDSS	
miRNA	r	*p*-Value	r	*p*-Value
hsa-miR-1973	−0.439	0.028	−0.489	0.013
hsa-miR-223-3p	0.399	0.048	0.481	0.015
hsa-miR-24-3p	0.447	0.025	0.464	0.019
hsa-miR-340-3p	0.424	0.035	0.482	0.015
hsa-miR-424-5p	0.449	0.025	0.546	0.005
hsa-miR-4466	−0.537	0.006	−0.507	0.010
hsa-miR-4485-5p	−0.430	0.032	−0.500	0.011
hsa-miR-6090	−0.435	0.030	−0.456	0.022
hsa-miR-6126	−0.430	0.032	−0.488	0.013

## Data Availability

MiRNAs raw and processed data are publicly available from the Gene Expression Omnibus database with the GSE230064 accession.
